# High doses of highly pathogenic avian influenza virus in chicken meat are required to infect ferrets

**DOI:** 10.1186/1297-9716-45-60

**Published:** 2014-06-03

**Authors:** Kateri Bertran, David E Swayne

**Affiliations:** 1 Exotic and Emerging Avian Viral Diseases Research Unit, USDA-ARS, 934 College Station Rd, 30605 Athens, GA, USA

## Abstract

High pathogenicity avian influenza viruses (HPAIV) have caused fatal infections in mammals through consumption of infected bird carcasses or meat, but scarce information exists on the dose of virus required and the diversity of HPAIV subtypes involved. Ferrets were exposed to different HPAIV (H5 and H7 subtypes) through consumption of infected chicken meat. The dose of virus needed to infect ferrets through consumption was much higher than via respiratory exposure and varied with the virus strain. In addition, H5N1 HPAIV produced higher titers in the meat of infected chickens and more easily infected ferrets than the H7N3 or H7N7 HPAIV.

## Introduction, methods, and results

Influenza virus infections in mammals are primarily respiratory centric with transmission via aerogenous droplets or contact with fomites [1]. However, H5N1 high pathogenicity avian influenza viruses (HPAIV) have caused fatal infections in large felids [2,3], domestic cats [4-6], and other carnivorous mammalian species [7-11] through consumption of infected bird carcasses or meat. In addition, human cases were reported following consumption of raw duck blood and organs [12] or after aspiration of exudate and blood from the upper respiratory tract of infected cocks [13].

The ferret model has been established for studying the pathogenicity and transmissibility of influenza viruses following respiratory and conjunctival routes of exposure [14]. Some H5N1 HPAIV can cause severe, fatal disease in ferrets after intranasal or intratracheal inoculation, mainly involving the respiratory tract with occasional virus strain specific systemic spread [15,16]. In addition, consumption of H5N1 HPAIV-infected chicken meat by ferrets has caused respiratory, gastrointestinal, and/or systemic disease depending on the virus strain and route of exposure [17]. However, little is known about the dose of virus required and the diversity of HPAIV subtypes that can cause infections following consumption of infected meat, which may be considered the natural exposure route in non-human carnivorous or omnivorous mammals. With the aim to answer these questions, ferrets were exposed to different HPAIV (H5 and H7 subtypes) through consumption of infected chicken meat.

The objective of Experiment 1 was to determine the ferret mean infectious (FID_50_) and lethal (FLD_50_) doses of two H5N1 HPAIV through consumption of infected meat: A/Whooper swan/Mongolia/244/05 (Mong/05) and A/Vietnam/1203/04 (VN/04). Previously, these same viruses were shown to infect ferrets following consumption of meat containing high virus concentrations (10^9.5^ EID_50_) [17]. Ferrets of 17–21 weeks-of-age were determined to be H5-seronegative by hemagglutinin inhibition (HI) and virus neutralization (VN) assays [18]. However, all animals possessed HI antibody titers to human H3N2 influenza A virus (A/Hiroshima/52/05), and 66% had HI antibody titers to human H1N1 influenza A virus (A/New Caledonia/20/99). For each virus, nine ferrets were divided into 3 groups (*n* = 3/group) representing a low, medium, or high exposure dose. Ferrets were fed breast meat (*pectoralis thoracicus* and *supracoracoideus*) collected from chickens 24 h after intranasal inoculation with Mong/05 or VN/04. Prior, for each virus three groups of meat (low, medium, and high) were classified based on virus concentration, and each meat group was given to the corresponding ferret group. Therefore, 30 g of specific titred infected meat was offered to each ferret individually. The dose consumed per ferret was calculated taking into account the virus concentration in the meat and the amount of meat consumed. Each of three individual ferrets received 10^4.2^ (low dose), 10^6.8^ (medium dose) or 10^9.2^ (high dose) mean egg infectious doses (EID_50_) of Mong/05, or 10^4.3^ (low dose), 10^6.9^ (medium dose) or 10^9.6^ (high dose) EID_50_ of VN/04. Ferrets were monitored for clinical signs and mortality. Body weight measurements and nasal washes for virus isolation were taken at 0, 3, 7, and 14 days post-challenge (dpc). Necropsy was performed on dead animals and the following tissues were collected for histologic examination: nasal cavity, lung, pharyngeal tonsil, esophagus, duodenum, pancreas, cecum, rectum, liver, spleen, kidney, heart, and brain. At 14 dpc, the remaining ferrets were bled and euthanized.

**Figure 1:**
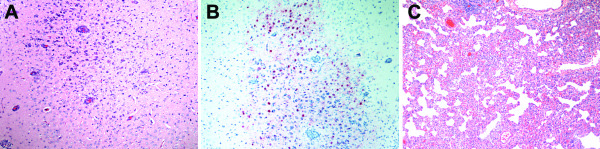
**Histopathologic changes in ferrets fed chicken meat infected with HPAIV (original magnification, ×100). (A)** Encephalomalacia, gliosis, and perivascular cuffings **(B)** with positive (brown) IHC staining of neurons and glial cells in a VN/04 infected ferret, 7 dpc. **(C)** Bronchiolar epithelial degeneration and necrosis, intraluminal cellular debris, and mild lymphocytic infiltration in an Egypt/07 infected ferret, 4 dpc.

None of the ferrets that consumed Mong/05 infected meat died but seven of nine became infected based on seroconversion as measured in VN test (Table 1). One high dose ferret had reduced activity, and one medium dose ferret demonstrated a weight loss of 25.3% from 7 to 14 dpc. All the other ferrets gained weight over the experiment (data not shown). None of the ferrets had virus recovery from nasal washes at any time point (data not shown). The FID_50_ was 10^4.9^ EID_50_, while the lack of deaths made FLD_50_ > 10^9.2^ EID_50_ (Table 1). By contrast, two ferrets that consumed high doses of VN/04 in infected meat died on 7 dpc, and two additional ferrets, one medium and one high dose exposed, became infected based on seroconversion (Table 1). The FID_50_ was 10^7.5^ EID_50_ and the FLD_50_ was 10^8.9^ EID_50_ (Table 1). The two ferrets that died experienced 25.7% and 11.7% of weight loss, had virus recovery from nasal washes only at 7 dpc (10^1.7^ and 10^1.5^ mean tissue culture infectious dose (TCID_50_)/mL), exhibited listlessness and ataxia, and had severe respiratory and systemic lesions including bronchial edema; severe multifocal encephalomalacia with accompanying gliosis, lymphohistiocytic perivascular cuffings and scattered vasculitis (Figure 1A), and lymphocytic infiltrates in ependymal cells of the ventricles; moderate pancreatic necrosis; and severe hepatic necrosis with periportal histiocytic infiltrates. Viral antigen was detected in the brain (neuropil, neurons, and glial cells) (Figure 1B), liver (Kupffer cells), and pancreas (acinar cells) and was associated with necrotic and inflammatory lesions. None of the surviving ferrets had virus recovery from nasal washes (data not shown), and only the high dose ferret that survived experienced a 16.6% of weight loss over the experiment.

**Table 1:** **Morbidity, mortality, and serological data from ferrets fed different quantities of H5N1 HPAIV in infected chicken meat to determine ferret mean infectious (FID**_**50**_**) and lethal (FLD**_**50**_**) doses**

**Virus group, dose (EID**_**50**_**)**	**Morbidity**	**Mortality**	Neutralizing antibody titers (14 dpc)^*^	**ID**_**50**_^**†**^**/LD**_**50**_^**‡ **^**(log**_**10 **_**EID**_**50**_**/0.1 mL)**
Mong/05				4.9/>9.2
Low (10^4.2^)	0/3	0/3	1/3 (113)	
Medium (10^6.8^)	1/3	0/3	3/3 (35, 226, 453)	
High (10^9.2^)	1/3	0/3	3/3 (320, 453, 2560)	
VN/04				7.5/8.9
Low (10^4.3^)	0/3	0/3	0/3	
Medium (10^6.9^)	0/3	0/3	1/3 (226)	
High (10^9.6^)	3/3	2/3	1/1 (394)	

dpc, days post-challenge; Mong/05, A/Whooper swan/Mongolia/244/05; VN/04, A/Vietnam/1203/04.

^*^Number positive/total of ferrets that seroconverted at 14 dpc based on virus neutralization (VN). In parenthesis, individual neutralizing antibody titers (<20 were considered negative).

^†^ID_50_, mean infectious dose; based on seroconversion by VN.

^‡^LD_50_, mean lethal dose; based on mortality.

The objective of Experiment 2 was to determine if other H5N1 (North Africa and Middle East) and H7 HPAIV (Europe and North America) could produce similar infections and disease from consumption of infected meat. Chickens were intranasally inoculated with a lethal dose of one of four HPAIV: A/chicken/Egypt/9402-NAMRU3HK213/07 (H5N1) (Egypt/07) (10^5.3^ EID_50_), A/Iraq/NAMRU3-207/06 (H5N1) (Iraq/06) (10^5.7^ EID_50_), A/chicken/Canada/314514-2/04 (H7N3) (Canada/04) (10^7.1^ EID_50_), and A/chicken/Netherlands/219/03 (H7N7) (Neth/03) (10^6.9^ EID_50_). Despite differences in the inoculated doses, all birds died on 2 dpc with high concentrations of virus present in Egypt/07 (10^9.0–9.2^ EID_50_/30 g) and Iraq/06 (10^10.4^ EID_50_/30 g) infected breast meat, and lower concentrations of virus present in Canada/04 (10^7.3–7.6^ EID_50_/30 g) and Neth/03 (10^7.5–7.8^ EID_50_/30 g) infected breast meat. Ferrets of 17–21 weeks-of-age were determined to be H5-and H7-seronegative by HI and VN. However, all animals possessed HI antibody titers to human H3N2 influenza A virus (A/Hiroshima/52/05). Four ferrets were fed 30 g of meat obtained from the infected chickens at doses listed above. Ferrets were monitored for clinical signs and mortality. Temperatures were recorded daily using subcutaneous probes. Body weight measurements, nasal washes, and rectal swabs were taken at 0, 1, 2, 4, 7, 10, and 14 dpc. At four and 14 dpc, two ferrets per virus were bled and euthanized, and tissues were collected for histologic examination.

None of the ferrets challenged with Egypt/07 died, but both ferrets euthanized at 14 dpc were infected based on seroconversion (Table 2). One ferret had reduced activity, 12% loss in weight, and moderate meningoencephalitis with no lesion-associated viral antigen when necropsied at 14 dpc. All four ferrets had respiratory lesions including bronchiolar epithelial necrosis with edema and congestion (4 dpc) (Figure 1C), or peribronchiolar lymphocytic infiltrates (14 dpc) with no positive IHC staining. None of the ferrets had an elevated temperature or virus recovery from nasal washes (data not shown). With Iraq/06, one ferret died at 3 dpc with an elevated temperature, and both ferrets euthanized at 14 dpc were infected based on seroconversion (Table 2). All the ferrets had mild interstitial pneumonia, the one that died also bronchiolar epithelial necrosis and peribronchiolar lymphocytic infiltrates. Among nasal washes and rectal swabs, virus was only recovered from the rectal swab of one ferret at 4 dpc (10^1.75^ TCID_50_/mL). Canada/04 exposed ferrets lacked mortality, but both ferrets euthanized at 14 dpc were infected based on seroconversion (Table 2). One ferret had reduced activity and an elevated temperature at 2 dpc. At 4 dpc, the two euthanized ferrets had mild interstitial pneumonia, which was most similar to the respiratory pathogenicity of non-Asian and Mong/05 H5N1 HPAIV. None of the ferrets had weight loss or virus recovery from nasal washes or rectal swabs (data not shown). None of the Neth/03 exposed ferrets became infected based on the lack of mortality and seroconversion (Table 2), lack of elevated temperature and weight loss, and lack of virus recovery from nasal washes or rectal swabs (data not shown).

**Table 2:** Individual serological data at 14 dpc from ferrets fed chicken meat infected with four different HPAIV

**Virus group, #ferret**	Neutralizing antibody titers (14 dpc)^*^
Egypt/07 #1	1810
Egypt/07 #2	905
Iraq/06 #1	1280
Iraq/06 #2	788
Canada/05 #1	35
Canada/05 #2	1114
Neth/03 #1	<20
Neth/03 #2	<20

dpc, days post-challenge; Egypt/07, A/chicken/Egypt/9402-NAMRU3HK213/07 (H5N1); Iraq/06, A/Iraq/NAMRU3-207/06 (H5N1); Canada/05, A/chicken/Canada/314514-2/05 (H7N3); Neth/03, A/chicken/Netherlands/219/03 (H7N7).

^*^Based on virus neutralization (VN) (<20 were considered negative).

## Discussion

Previous studies have demonstrated infection in ferrets by H5N1 viruses via intranasal exposure or consumption of infected meat. Ferrets intranasally inoculated (10^6^ EID_50_) or fed infected meat (10^9.5^ EID_50_) with Mong/05 lacked mortality but seroconverted [17] while respiratory and systemic lethal disease was reported following intranasal inoculation (10^6^ EID_50_) or oral consumption (10^9.5^ EID_50_) of infected meat with VN/04 [17]. Furthermore, Mong/05 was infectious at each of the intranasal doses from 10^1^ to 10^4^ TCID_50_[19], and VN/04 proved to cause severe systemic infection and mortality after intranasal doses as low as 10^1^ EID_50_[16,17,19]. The current study had moderate to high FID_50_ and FLD_50_ for infection via consumption of infected meat suggesting the dose of virus needed to infect and/or kill ferrets through consumption of infected meat is much higher than for the same virus via respiratory exposure.

In the current study, consumption of non-Asian H5N1 viruses caused primarily respiratory disease. However, the presence of mild meningoencephalitis without neurological signs or viral antigen detection in the brain of one Egypt/07 infected ferret suggests some extent of systemic spread with damage to the nervous system. Therefore, North African and Middle Eastern H5N1 HPAIV caused infection in ferrets through feeding meat containing high concentrations of HPAIV and produced primarily respiratory disease, being less lethal than Asian VN/04. Consumption of H7 HPAIV infected meat was less pathogenic or non-infectious for ferrets compared to H5N1 HPAIV, which may be explained by the difference in infection efficiency of H5 compared to H7 AIV. However, the 2–3 log_10_ lower concentration of H7 HPAIV produced in meat of infected chickens compared to H5N1 HPAIV could be responsible for mild pathogenicity (Canada/04) or even absence of infection (Neth/03) in ferrets. Whether a higher virus dose in meat would allow Neth/03 to infect through consumption warrants further study, although the inability to produce a high concentration of H7 HPAIV in meat may prevent testing the hypothesis. Supporting the infection efficiency hypothesis, both respiratory and ingestion exposures of Asian H5N1 HPAIV at 10^4^ TCID_50_ produced systemic virus replication with severe necrosis and inflammation in cats [20] while inoculation with the same dose of a 2003-Dutch-H7N7 HPAIV seemed to restrict replication to the respiratory tract [21]. Also, Neth/03 and other 2003-Dutch-H7N7 HPAIV were highly virulent and lethal in ferrets after intranasal inoculation of 10^7^ EID_50_, causing respiratory and neurological signs, and systemic lesions [22]. Collectively, these findings indicate that pathogenesis as to being respiratory, gastrointestinal or systemic may be highly dependent not only on the viral strain and inoculating dose, but also on the route of exposure, as previously suggested [17,23], with higher doses of HPAIV being required to produce infection via oral consumption of infected meat compared to respiratory exposure.

In conclusion, relatively high concentrations of H5N1 HPAIV are required to produce infection and death by consumption of infected meat in ferrets as compared to respiratory exposure. Ingestion of HPAIV-infected meat can produce infection that primarily involves the respiratory tract but can also spread systemically depending on both the virus strain and virus dose received. Although human infections by HPAIV through direct oral contact have been occasionally reported [12,13], airborne virus or contact with fomites is still considered the main route of exposure in human species [1].

## Competing interests

The authors declare that they have no competing interests.

## Authors’ contributions

KB analyzed the results and carried out the histopathological examinations. DES conceived the study and participated in its design, coordination, and implementation. KB and DES draft the manuscript. Both authors read and approved the final manuscript.
